# Investigation on the Performances of Esterified Waste Cooking Oil Rejuvenator and Recycled Asphalt

**DOI:** 10.3390/ma17194725

**Published:** 2024-09-26

**Authors:** Junhui Wang, Qunshan Ye, Lingyi Fan, Cheng Xie, Haobin Liu

**Affiliations:** 1 School of Traffic and Transportation Engineering, Changsha University of Science and Technology, Changsha 410114, China; 22201060242@stu.csust.edu.cn (J.W.); 21101030029@stu.csust.edu.cn (L.F.); 2 Guangxi Beitou Transportation Maintenance Technology Group Co., Ltd., Nanning 530201, China; xiecheng1357@163.com (C.X.); liuhaobin9203@163.com (H.L.)

**Keywords:** recycled asphalt, waste cooking oil, glycerol esterification, thermo-oxidative stability, anti-aging properties

## Abstract

Waste cooking oil (WCO) recycled asphalt is facing issues regarding insufficient thermal oxidation stability and aging resistance. In this research, glycerol esterification was adopted to pretreat WCO, and the consequences of this treatment on the aging resistance and thermal stability of WCO were analyzed. The impacts of varying levels of esterification of WCO on the high-temperature, low-temperature performances, fatigue properties, and aging resistance of recycled asphalt were investigated. Furthermore, the mechanisms of regeneration and the anti-aging of deeply esterified WCO recycled asphalt were revealed by Fourier transform infrared spectroscopy (FTIR) and gel permeation chromatography (GPC) tests. The results indicated that variations in the physical properties of WCO during the aging process were reduced, and its aging resistance was improved following glycerol esterification therapy. The initial thermal decomposition temperature was increased by approximately 115 °C, which resulted in the enhancement of thermal stability significantly. Recycled asphalt obtained from deeply esterified WCO exhibited superior high-temperature, low-temperature performances, and fatigue properties. Moreover, the thermal oxidation stability and aging resistance of recycled asphalt with deep-esterified WCO could be promoted by reducing the oxidation and volatilization of light components during the aging process, with the complex modulus ageing resistance index decreasing by 13.27% and the phase angle ageing resistance index increasing by 14.71%.

## 1. Introduction

According to the statistical bulletin on the development of the transportation industry published by the Ministry of Transport of China in 2022, 99.9% of China’s gross roadway miles, which amount to over 5.3 million kilometers, are currently undergoing maintenance. Annually, approximately 300 million tons of reclaimed asphalt pavement (RAP) materials are generated during medium and major repair projects [1,2,3]. However, it is reported that less than 30% of pavement materials are recycled, which is significantly lower than the over 80% recycling rates prevalent in developed countries, resulting in enormous resource waste and ecological harm [4,5]. Consequently, it is acknowledged that recycling RAP on an extensive scale employing recycling technologies is an essential method to accomplish green construction in the highway transportation sector [6,7], motivate synergistic effects in reducing pollution and carbon emissions, as well as enable a comprehensive green transformation of social and economic development [8,9,10,11,12].

Existing research typically focuses on rejuvenating aging asphalt in RAP with low molecular weight rejuvenators to achieve objectives, including minimizing resource consumption, saving construction costs, and diminishing carbon emissions [13,14]. Based on research into the rheological properties and recycling mechanisms of aged asphalt, it has been discovered that the addition of light oils can enhance the relative contents of saturated and aromatic fractions in aged asphalt, adjust the colloidal structure, as well as restore the crack resistance, water damage resistance, and durability of the aged asphalt, facilitating it to satisfy road performance requirements [15,16]. Asphalt rejuvenators are currently classified into two main categories: petroleum-based and bio-based. However, petroleum-based rejuvenators (including aromatic oil extracts and naphthenic oils) possess deprived high-temperature stability, resulting in insufficient anti-aging performance of the recycled asphalt, as well as high material costs and non-renewability, which contradict the concepts of sustainability [17]. Consequently, the pursuit of a low-cost, environmentally friendly, and significantly effective asphalt rejuvenator is considered crucial for achieving the rational and efficient recycling of asphalt [18]. It has been estimated that the amount of WCO formed globally each year is enormous, with China alone producing up to 20 million tons of WCO each year, whereinthe major components of WCO are comparable to the lightweight components lost during the aging process of asphalt [19,20,21]. Moreover, compared to petroleum-based rejuvenators, a lower dosage of WCO is adequate to convey aged asphalt’s penetration, softening point, and viscosity back up to virgin asphalt levels [22,23,24,25]. The dual waste utilization of RAP and WCO has been successfully established through the development and promotion of WCO recycled asphalt, offering significant benefits in terms of the economy, environment, and resources.

Nonetheless, the intricate origins of WCO have resulted in problems, including uneven quality and erratic rejuvenator effectiveness [26,27]. WCO rejuvenators with high acid values include substantial levels of free unsaturated fatty acids, which are susceptible to volatilization and oxidation, hastening the aging process and performance degradation of recycled asphalt [28,29]. As of this moment, dehydration and simple impurity removal are the most prevalent methods to handle WCO [30], but the impact that fluctuations in WCO acid values have on the rejuvenator and how effectively it performs with recycled asphalt have not been given sufficient attention. Thus, in order to enhance the durability of recycled asphalt, it is imperative to investigate effective pretreatment techniques for WCO rejuvenators.

In this study, WCO has been pre-treated using glycerol esterification techniques, and the impacts of various esterification levels on the properties of rejuvenated asphalt have been examined, along with the performance outcomes of esterified WCO rejuvenators in terms of thermal oxidative stability. Additionally, the mechanism of action of esterified WCO rejuvenators has been explored through microscopic experiments, revealing how the performance of aged asphalt could be improved. This research not only establishes a relatively reliable method for optimizing the performance of WCO rejuvenator but also provides a theoretical basis for the widespread application of WCO recycled asphalt.

## 2. Materials and Methods

### 2.1. Materials

#### 2.1.1. Base Asphalt

The base asphalt is 70# A-grade road petroleum asphalt from Hunan Baoli Asphalt Co., Ltd. (Changsha, China), and the specific performance indicators are shown in Table 1.

#### 2.1.2. Waste Cooking Oil

The waste cooking oil (WCO) provided by a waste oil recovery company in a certain region of Shandong is dark brown, with an average molecular weight of 890. Before the experiment, contaminants were removed using a simple filtration method, eliminating the moisture and impurity content of the WCO to mitigate more than 1%. The results of its conventional physical attributes are displayed in Table 2.

### 2.2. Test Methods

#### 2.2.1. Pre-Treatment of WCO

**Figure 1:**
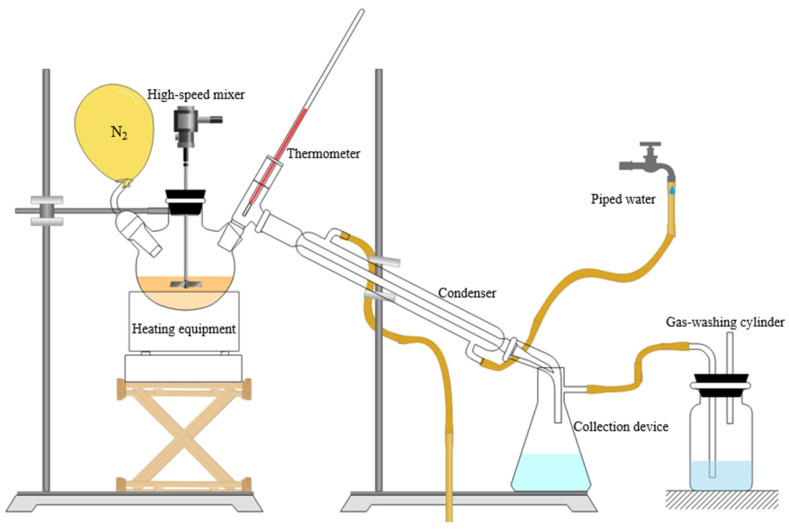
Esterification reaction test equipment.

In this study, WCO was pretreated using the glycerol esterification method, and the optimum test conditions were 240 °C, 60 min, 500 r/min, and an oil–alcohol molar ratio of 1.1:1. The glycerol esterification reaction test equipment is depicted in Figure 1. Nitrogen gas was released through a wash bottle holding the organic solvent after it passed from the vent through the liquid’s surface during the test. In this period, water vapor was produced, which was then condensed through a condensation tube and gathered in a collection device.

#### 2.2.2. Preparation of Aged Asphalt

Following ASTM D2872 [40], 35 g ± 0.5 g of virgin asphalt (VA) was aged for 85 min at 163 °C in a rotating thin-film oven (RTFOT) to generate short-term aged asphalt (SA). Subsequently, under ASTM D6521 [41], the collected short-term aged asphalt was placed in a pressure aging vessel (PAV) and aged for 20 h under conditions of 100 °C and 2.1 MPa pressure to obtain long-term aged asphalt (LA). The performance indicators of the virgin asphalt and the asphalt at different aging levels are shown in Table 3.

#### 2.2.3. Preparation of Recycled Asphalt

In order to investigate the impact of esterified WCO on the performance of recycled asphalt, three types of WCO were distinguished according to varying degrees of pretreatment: original WCO that was not treated (O-WCO), moderately esterified WCO (M-WCO) with a glycerol-to-fatty acid molar ratio of 0.5:1, and WCO that was deeply esterified (D-WCO), which satisfied the optimum pretreatment conditions. Referring to the optimal dosage range for restoring aged asphalt performance, these three rejuvenators with different degrees of esterification were added at a fixed dosage of 6% to 300g of long-term aged asphalt in molten form. Utilizing a high-speed shear mixer set to 2000 r/min for 30 min, the mixture was sheared to produce recycled asphalt, with the heating jacket maintained at a temperature of 135 °C ± 5 °C. Based on the esterification degree of the added rejuvenators, the recycled asphalts were named O-RA, M-RA and D-RA [42]. The acid value ranges of different degrees of esterified WCO rejuvenators and the corresponding names of the recycled asphalts are shown in Table 4.

#### 2.2.4. Determination of the Acid Value of WCO

According to ASTM D974, the hot ethanol technique was utilized to determine the acid value of WCO under various reaction circumstances. To neutralize free fatty acids, a 0.1 mol/L sodium hydroxide solution was titrated in a moderately boiling ethanol–WCO solution. The volume of the standard solution needed to reach the titration’s endpoint was measured to determine the acid value.

#### 2.2.5. Viscosity Measurement of WCO

A Brookfield rotational viscometer from the USA was used to determine the 60 °C rotational viscosity of the same mass (50 g ± 0.5 g) of O-WCO and D-WCO placed in a film oven at 163 °C after 2, 4, 6, and 8 h of thermo-oxidative aging treatment, and then the differences in the viscosity changes of the different rejuvenators were analyzed.

#### 2.2.6. Thermal Decomposition Test

The relationship between the thermal decomposition of WCO and temperature under a nitrogen atmosphere were obtained by thermogravimetry–derivative thermogravimetry (TG-DTG) simultaneous analyzer to determine the effect of glycerol esterification pretreatment on the thermo-oxidative stability of regeneration. We aimed for 800 °C as the target temperature, and we set the rate of temperature increase to 10 °C/min.

#### 2.2.7. Rheological Properties Test of Asphalt

The high-temperature stability, low-temperature cracking resistance, fatigue performance, and resistance to thermal-oxidative aging of recycled asphalt made from WCO at varying degrees of esterification were all assessed in this study through an Anton Paar MCR 302 rheometer via frequency scanning (temperature: 5, 10, 15, 28, 40, and 52 °C; frequency: 0.016 Hz~16 Hz), temperature scanning (temperature: 43 °C~82 °C; angular frequency: 10 rad/s; strain level: 6%), linear amplitude scans (temperature: 25 °C; strain level: 0.1%~30%), and multiple stress creep recovery tests (1. Stresses: 0.1 kPa, cyclicality: 20; 2. Stresses: 3.2 kPa, cyclicality: 10).

#### 2.2.8. Microstructure Testing of Asphalt

The functional group properties of WCO, aged asphalt, and WCO-recycled asphalt were examined using Fourier transform infrared spectroscopy (Nicolet iS50) from Thermo Fisher Scientific Inc., Carlsbad, CA, USA. with the wavelength range of 500 cm^−1^ to 4000 cm^−1^, which offers a qualitative analysis of the evolution of the microscopic structure during the recycling and aging processes of asphalt.

Gel permeation chromatography (PL-GPC50) was used to investigate the effects of the WCO rejuvenator on the components of aged asphalt, the aging mechanism of asphalt via molecular weight distribution, and the anti-aging performance of deeply esterified WCO-recycled asphalt. Tetrahydrofuran (THF) at a concentration of 2 mg/mL was chosen as the organic solvent. The elution procedure took place over 20 min, with a temperature control of 40 °C.

## 3. Results

### 3.1. Anti-Aging Properties of WCO Regenerations with Different Degrees of Esterification

**Figure 2:**
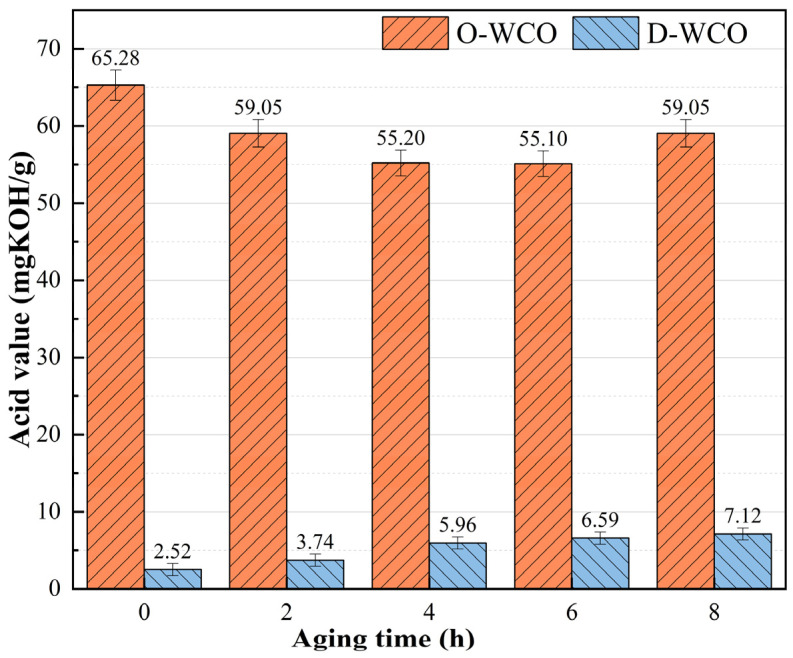
Variation of WCO acid value with varying esterification levels.

**Figure 3:**
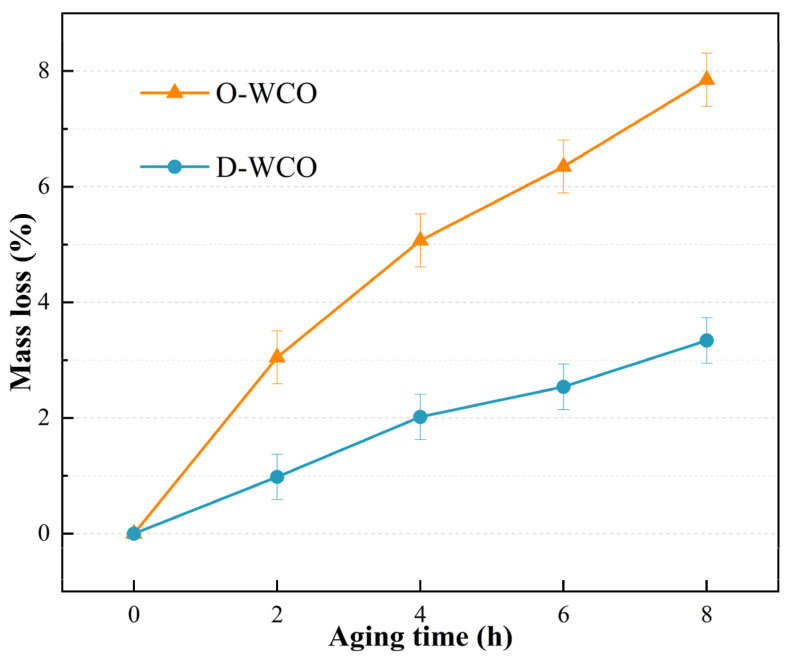
Variation of WCO mass loss rate with varying esterification levels.

**Figure 4:**
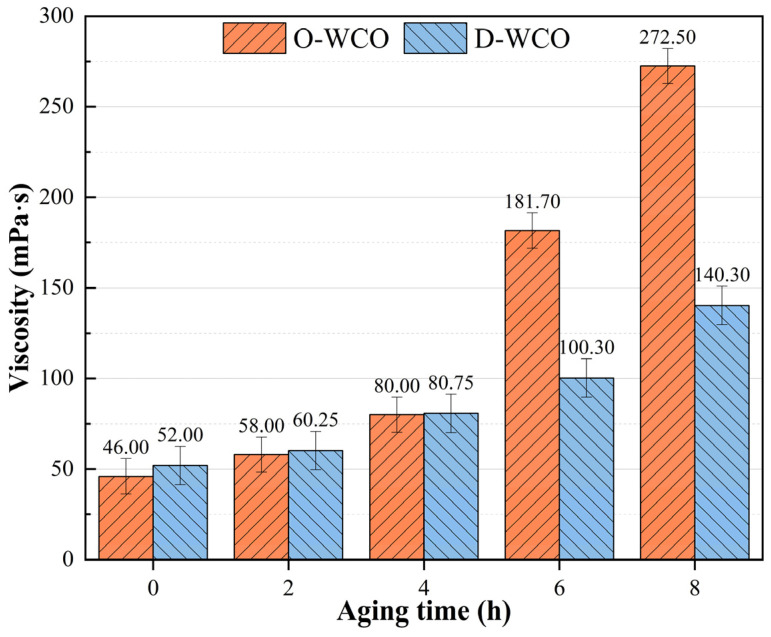
Variation of WCO viscosity with varying esterification levels.

The fluctuation patterns of the two WCOs’ three physical properties (mass, viscosity, and acid value) against the thermo-oxidative aging period are shown in Figure 2, Figure 3 and Figure 4, which show how the esterification treatment affects the WCO regenerator’s performance.

#### 3.1.1. Effect of Thermo-Oxidative Aging on Regenerants’ Acid Value

The initial acid value of D-WCO was almost 96% lower than that of O-WCO, as evidenced by the data in Figure 2, suggesting that glycerol esterification significantly decreased the acid value of WCO. A modest increase in the acid value of D-WCO was seen with an increase in the thermo-oxidative aging duration. This could be attributed to the conversion of readily oxidizable functional groups in the oil to carboxyl groups during the thermo-oxidative aging process [43]. However, despite the increase in acid value, it was maintained at a low level compared to O-WCO, showing excellent performance stability.

The initial acid value for O-WCO was high, but as the duration of thermal oxygen treatment increased, it showed a slightly decreasing tendency. This may be due to the fact that it contained high levels of thermally less stable free fatty acids that decompose at high temperatures, and the amount of decomposition exceeds the amount of unsaturated fatty acids produced during the oxidation process, thus slightly lowering the acid value. When consumed to a certain extent, the acid value slowly increased as the oxidation process proceeded.

#### 3.1.2. Effect of Thermo-Oxidative Aging on Regenerants’ Rate of Mass Loss

The mass loss of D-WCO and O-WCO during the thermal oxidative aging process are shown in Figure 3. According to the research, both oil samples had mass changes during thermal-oxidative aging; however, O-WCO shows a significantly higher mass loss rate than D-WCO. This indicates that during the glycerol esterification reaction, some of the compounds such as alcohols, aldehydes, ketones and heterocycles, which are readily volatile and decomposable under thermal oxidative conditions, were consumed, leading to a more stable performance of the pre-treated oil and effectively improving the rejuvenator’s resistance to thermal–oxidative aging.

#### 3.1.3. Effect of Thermo-Oxidative Aging on Regenerants’ Viscosity

Figure 4 illustrates how the viscosity of both oil samples increased as the thermal oxidative aging period increased. This phenomenon was primarily attributed to the presence of a large number of unsaturated fatty acids (such as linoleic acid and alpha-linolenic acid) in the regenerants, whose α-position carbon–hydrogen bonds are easily attacked, promoting free radical chain reactions that generate oxidation and polymerization products, which caused a significant increase in viscosity. When exposed to thermal–oxidative conditions, D-WCO exhibited a more modest increase in viscosity than O-WCO, suggesting that the esterification reaction can effectively postpone the aging process of the WCO rejuvenator.

### 3.2. Pyrolysis Characteristics of WCO Regenerants with Different Degrees of Esterification

**Figure 5:**
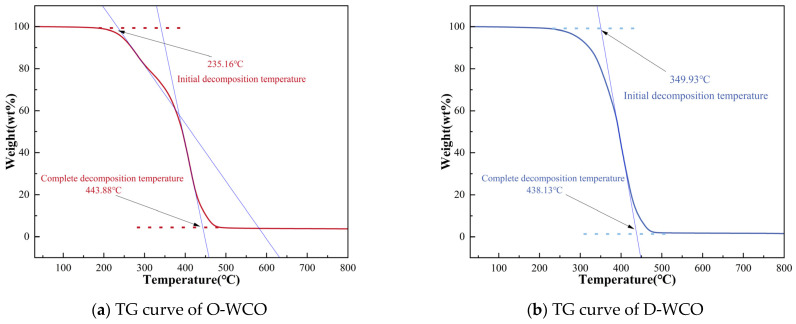

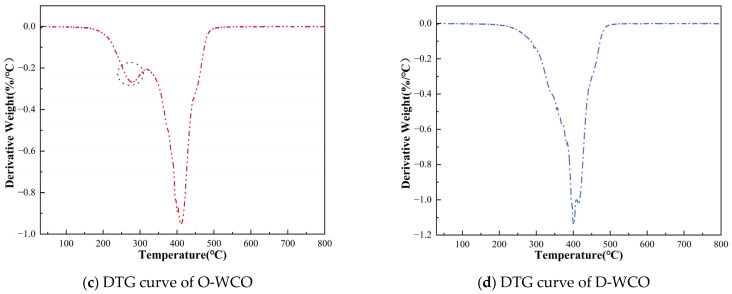
TG and DTG curves of WCO at varying esterification degrees.

Good pyrolysis characteristics help ensure the stability of regenerant performance, as regenerants are prepared and used under high temperature conditions. The present investigation employed the thermogravimetric analysis (TGA) technique to examine the regenerant’s thermal decomposition behavior. Additionally, thermogravimetric (TG) curves and derivative thermogravimetry (DTG) curves were plotted to visualize the variations in the WCO mass retention and weight loss rate with temperature during the warming process (Figure 5).

The thermal decomposition of WCO could be separated into three major stages, as evidenced by the trends in the mass retention and mass loss rates of the regenerant. The first stage (<350 °C) was the initial reaction phase, where the mass loss of the rejuvenator was primarily due to the volatilization of lighter components and small molecular oils in WCO under high-temperature conditions, resulting in a low weight loss rate. The second stage (350 °C–450 °C) was the reaction phase, in which the main components of the rejuvenator began to combust and decompose, reaching the highest weight loss rate. The third stage (>450 °C) was the end of the reaction phase; during this stage, both the mass retention and weight loss rate curves of the rejuvenator exhibited a more stable trend, indicating that the main components of the rejuvenator were largely burnt out.

The main differences between O-WCO and D-WCO’s thermal breakdown characteristics were evident in the first stage, with the second and third stages showing significant similarities. O-WCO began to decompose at 235 °C, whereas D-WCO’s initial decomposition temperature was boosted to more than 340 °C, representing a 48.81% increase. In comparison to D-WCO, the DTG curve of O-WCO had one small mass loss rate peak in the first stage, which indicates that the glycerol esterification almost completely consumed the components of WCO that were susceptible to volatilization under pyrolysis conditions and effectively improved the thermal stability of the WCO regenerant below 350 °C.

Based on kinetic equations and the Coats–Redfern method, a kinetic model of the thermal decomposition reaction of two oil samples was created. Kinetic parameters, such as the activation energy *E* and fingering front factor *A*, were then calculated to serve as a reference for the quantitative investigation of the regenerant’s thermal stability [44]. The outcomes are displayed in Table 5.

From the perspective of activation energy, D-WCO exhibited a higher activation energy *E*, indicating that the energy barrier for the thermal decomposition reaction was greater, making the decomposition process less likely to occur compared to O-WCO. Considering the change in enthalpy, the enthalpy change value of D-WCO was significantly greater than that of the untreated oil, suggesting that a larger amount of reaction heat was required for the thermochemical decomposition of WCO after glycerol esterification treatment, which reinforces the conclusion that the thermal decomposition reactions were less likely to occur. This supports the findings of the activation energy analysis and provides more evidence that the esterification reaction can significantly improve the thermal stability of WCO.

### 3.3. Performance Analysis of WCO Recycled Asphalt with Different Acid Values

#### 3.3.1. High-Temperature Stability

**Figure 6:**
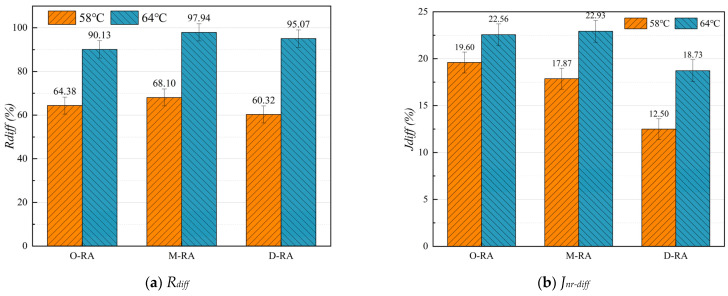
Effects of regenerants with different esterification degrees on stress sensitivity coefficients.

A multiple stress creep recovery (MSCR) test was applied to investigate the rheological properties of the recycled asphalts, calculate the stress sensitivity coefficients R_diff_ and J_nr-diff_ of the various recycled asphalts, and analyze the impact of the esterification degree of waste cooking oil (WCO) on the high-temperature stability of the recycled asphalt. From Figure 6, it can be seen that the R_diff_ and J_nr-diff_ values for the three types of recycled asphalts at 64 °C were all higher than those at 58 °C, indicating greater viscoelastic changes and higher stress sensitivity at elevated temperatures; this observation is consistent with the phenomenon of rutting in asphalt under high-temperature service conditions. Comparing the recycled asphalt prepared with the same dosage of three different esterification levels of reclaimers, D-RA showed the lowest stress sensitivity at the same temperature, indicating that the esterification treatment can improve the high temperature stability performance of this recycled asphalt.

#### 3.3.2. Low-Temperature Crack Resistance

**Figure 7:**
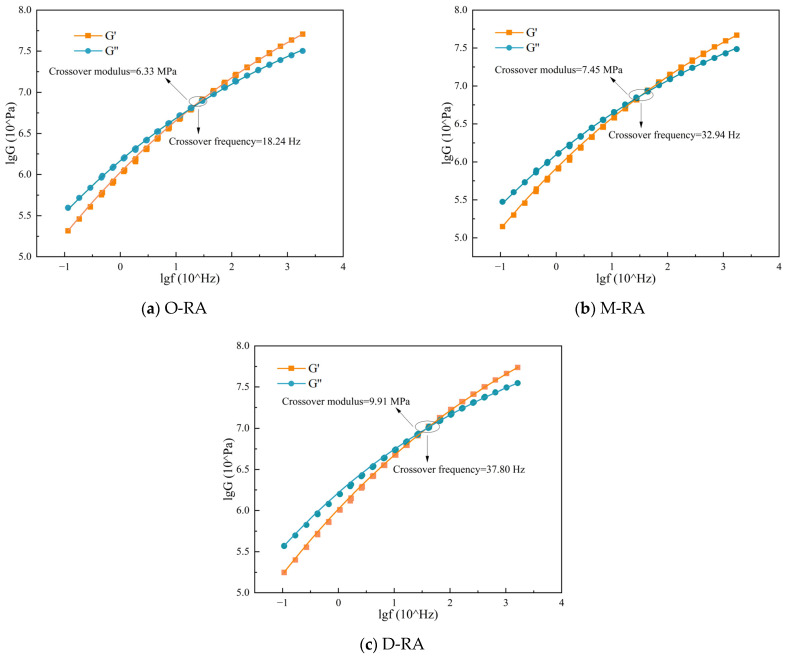
Cross-modulus and cross-frequency values of WCO recycled asphalts with different esterification degrees.

The viscoelastic behavior of the asphalts at varying loading frequencies was investigated with the dynamic shear rheometer’s frequency scanning mode. The asphalt storage modulus G′ and loss modulus G″ occurring in the high frequency range were determined utilizing the time–temperature equivalency concept, and the corresponding logarithmic curves were plotted. When the two lines connect, the cross-modulus and cross-frequency can be found, allowing for the evaluation of the asphalt material’s crack resistance [45,46], as illustrated in Figure 7. Higher crossover frequencies were associated with lower asphaltene content, as the values demonstrated a strong association with asphaltene content [47]. D-RA had the highest crossover frequency, followed by M-RA and O-RA. This demonstrates that D-RA has better low-temperature cracking resistance, since it has lighter components and less asphaltene. The low-temperature cracking resistance of WCO-recycled asphalt improved as the esterification degree rose, according to an analysis of the crossover frequency trend.

#### 3.3.3. Fatigue Property

**Figure 8:**
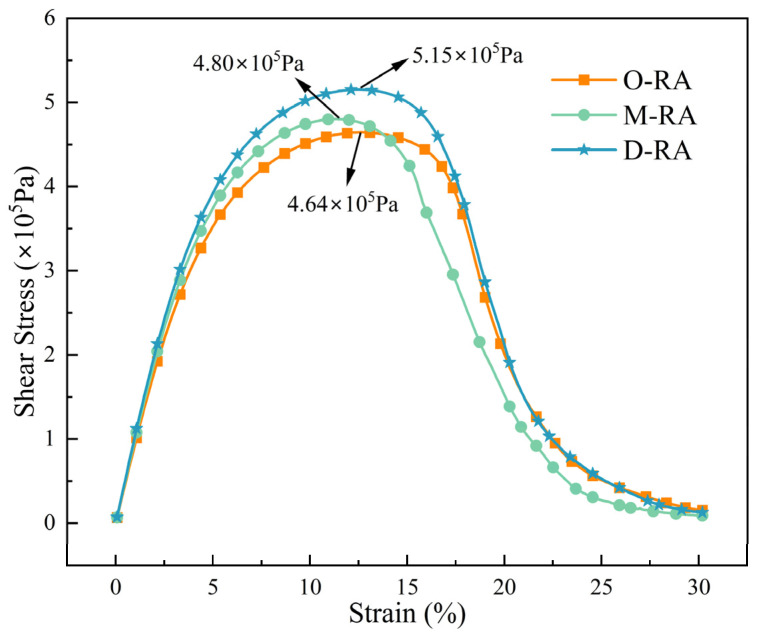
The stress–strain curves of WCO recycled asphalt with different degrees of esterification.

To assess the fatigue performance levels of the asphalts, the Linear Amplitude Sweep (LAS) test method, as outlined in AASHTO TP 101-12, was utilized in this study. Figure 8 displays the stress–strain curves of the recycled asphalts with varying acid values. When compared to the other two rejuvenators, D-RA showed the highest peak shear stress and maximum yield stress, indicating greater fatigue performance. Additionally, D-RA exhibited a significantly broader half peak than M-RA and O-RA, which indicates that it has the longest fatigue life. In conclusion, the oil components treated with esterification positively influenced the fatigue performance of recycled asphalt, improving the yield stress and extending the fatigue life.

#### 3.3.4. Thermo-Oxidative Aging Resistance

**Figure 9:**
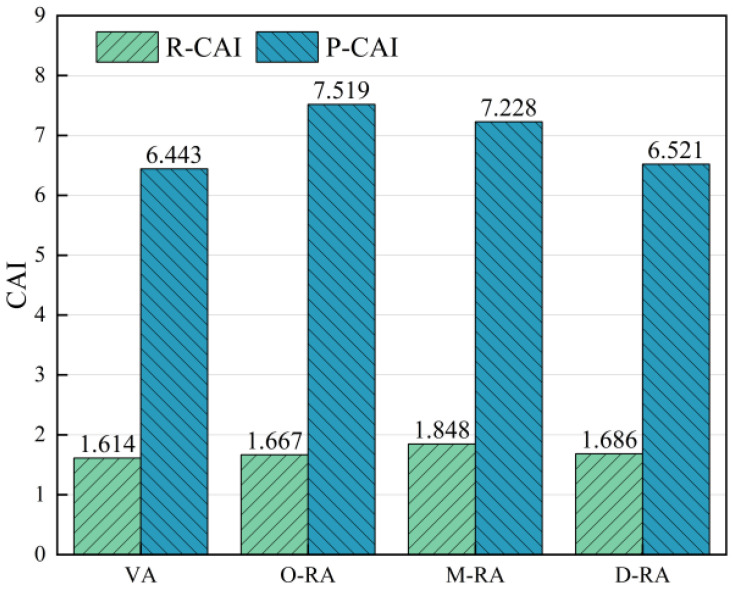
Complex modulus values anti-aging index.

**Figure 10:**
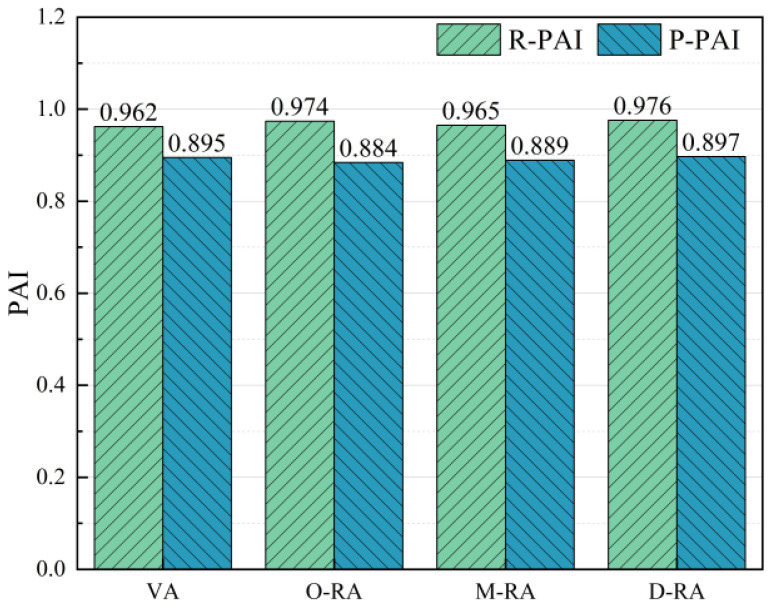
Phase angle values anti-aging index.

Following short-term and long-term aging of the base asphalt and three types of recycled asphalts, temperature scans of the asphalt specimens were conducted. The complex modulus aging index (CAI) and phase angle aging index (PAI) were then utilized as the evaluation indexes of the asphalts’ resistance to aging. For clarity of presentation, the short- and long-term aging resistance indexes of the complex modulus were denoted as R-CAI and P-CAI, and the short- and long-term aging resistance indexes of the phase angles as R-PAI and P-PAI. As shown in Figure 9 and Figure 10, the complex modulus values rose, while the phase angles decreased as the asphalt aged, so the lower CAI and higher PAI values indicate better asphalt resistance to aging.

From the perspective of CAI, the R-CAI values of the WCO recycled asphalt with different degrees of esterification were quite similar and slightly higher than the short-term anti-aging index values of the base asphalt. This indicates that pretreatment has less of an effect on recycled asphalt’s resistance to short-term aging. This could be as a result of less volatilization of the lightweight components under short-term aging circumstances of 163 °C and 85 min, leading to a closer change in the complex modulus. In contrast, the base asphalt has fewer lightweight components and a smaller molecule oil content than recycled asphalt, so it showed better resistance to short-term aging. D-RA had the lowest P-CAI among the three types of recycled asphalt, which was comparable to the base asphalt according to the trend of P-CAI changes in the recycled asphalt. This suggests that glycerol esterification treatment can enhance the thermal stability of the reclaimer, maintain the proportion of lightweight components of the reclaimed asphalt, and improve the resistance to long-term aging.

The effect of aging on the percentage of viscoelastic materials in asphalt was represented by the PAI values. D-RA exhibited the lowest phase angle loss during age under the identical regenerant dosing conditions, with M-RA and O-RA showing the greatest phase angle reductions in that order. This indicates that among rejuvenators with varying degrees of esterification, the deeply esterified rejuvenator has the most significant effect on slowing down the changes in the proportion of viscoelastic components in asphalt during the aging process. In addition to providing support for the lightweight components, the pre-treated rejuvenator also improved the thermo-oxidative stability of the recycled asphalt, reduced the loss of volatile components during thermo-oxidative aging, and increased the performance of the recycled asphalt retention capacity.

### 3.4. Microstructural and Mechanistic Analysis of Recycled Asphalt

#### 3.4.1. Analysis of WCO Recycled Asphalt’s Functional Group Changes and Antioxidant Properties

**Figure 11:**
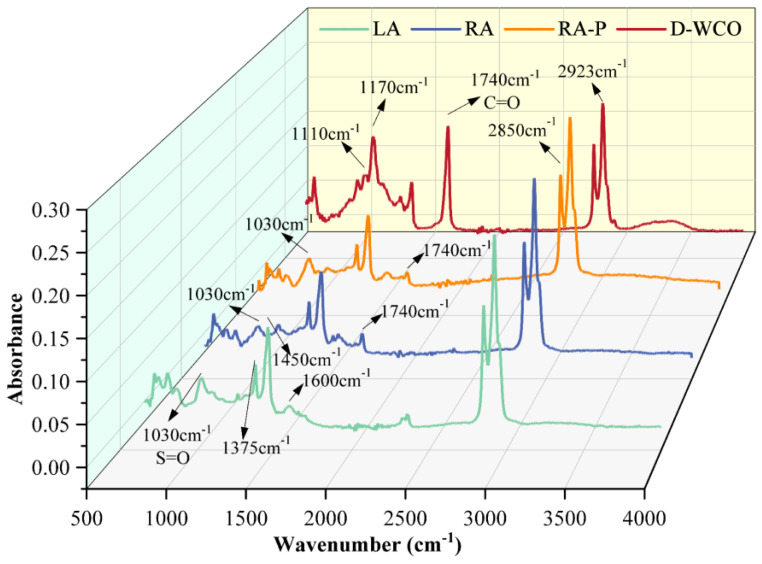
Infrared spectrogram of D-WCO and different asphalts.

The infrared spectral tests were conducted on long-term aged bade asphalt, recycled asphalt, long0term aged recycled asphalt, and D-WCO using a Fourier transform infrared spectrometer (Nicolet iS50) with a wavelength test range from 500 cm^−1^ to 4000 cm^−1,^ and the infrared spectrogram is shown in Figure 11. LA stands for long-term aged matrix asphalt, RA denotes recycled asphalt, and RA-P refers to long-term aged recycled asphalt.

Stretching vibration peaks were seen in the spectra for methyl groups (2093 cm^−3^), methylene groups (2850 cm^−3^), and benzene rings (1700 cm^−3^) for LA, RA, RA-P, and D-WCO. Furthermore, deformation vibration peaks, which belong to methylene groups, were visible at 1375 and 1450 cm^−1^. Additionally, D-WCO showed characteristic peaks at 1740 cm^−1^, 1375 cm^−1^, and 1450 cm^−1^, indicating that the functional group contains an ester group. When D-WCO was added to LA, a new ester carbonyl (-C=O-) absorption peak appeared in RA. In the recycled asphalt, besides the classic peaks of D-WCO and LA, no additional new absorption peaks were identified. This indicates that the physical co-mingling between WCO and asphalt was predominant and that no chemical reaction occurred or that the reaction was weak. The strength of the sulfoxide (-S=O-) absorption peak at 1030 cm^−3^ in LA decreased from 0.06 to around 0.04 after D-WCO was added, and then it increased to approximately 0.05 after long-term aging, which was still less than the initial value. This indicates that the sulfoxide intensity was effectively reduced by the addition of D-WCO and that the esterification reaction also somewhat increased the oxidative resistance of the D-WCO recycled asphalt.

#### 3.4.2. Laws of Molecular Evolution during Aging of Base Asphalt and Recycled Asphalt

**Figure 12:**
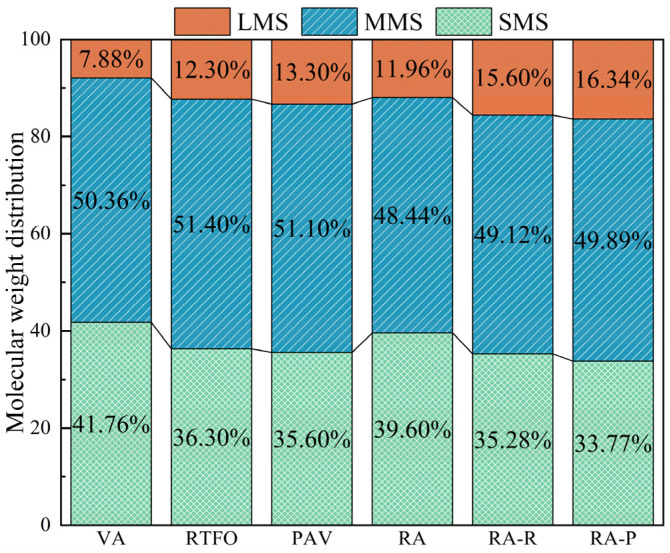
Effect of aging on molecular weight distribution.

The molecular weight and distribution properties of base asphalt, short-term aged asphalt, long-term aged asphalt, recycled asphalt, and recycled asphalt with varying degrees of aging were examined in this study using gel chromatography (PL-GPC50). The relative proportions of molecular weights are displayed in Figure 12.

By introducing the rejuvenator, the proportion of large molecular weight substances (LMSs) could be reduced while diluting the aged asphalt according to its rich light components, thereby adjusting the molecular weight distribution. In addition to the diluting effect, the depolymerization of asphalt dimers was also included in the rejuvenator’s mechanism of action, which further reduces the aggregation of large molecules [48]. By reducing the proportion of large molecular weight compounds (LMSs) and boosting the amounts of small molecular weight substances (SMSs) and medium molecular weight molecules (MMSs), this process efficiently optimizes the molecular composition of aged asphalt. During the aging process of the asphalt materials, the proportion of LMSs increased while the proportion of SMSs decreased, revealing the impact of aging on the molecular composition of the asphalt. This phenomenon could be attributed to the volatilization and oxidation of light small molecular components during the aging stage, leading to a decrease in small molecular quantities or their transformation into medium and large molecular weight substances [49]. Since WCO rejuvenators primarily achieve the regeneration of aged asphalt by supplementing a large number of light components, the aging process of rejuvenated asphalt is also marked by the molecular change phenomena.

The number-average molecular weight (Mn), weight-average molecular weight (Mw), and polydispersity index (PDI) of asphalt at different aging degrees are listed in Table 6. The volatilization of oil components after aging leads to an increase in the Mn and a decrease in the Mw of the composite rejuvenated asphalt; however, the data presented show a trend of increasing Mw, indicating that aggregation occurs between asphaltene molecules after asphalt aging, resulting in an increase in the large molecular content [50].

The polydispersity index (PDI) in the GPC results of asphalt were correlated with the high-temperature performance outcomes of the asphalts. As the PDI increased, the high-temperature performance improved [51]. With the increase in the aging degree, the PDIs of the asphalts also increased, and the PDIs of the recycled asphalts at each stage were greater than that of the base asphalt. This indicates that following pretreatment, the WCO eliminated some tiny molecules with poor thermal stability, producing a more stable and effective light component supplement for the aged asphalt that reduced volatility and improves the recycled asphalt’s performance at high temperatures.

## 4. Conclusions

(1) After 2, 4, 6, and 8 h of thermal oxidation aging, the mass, viscosity, and acid value changes of D-WCO were all smaller than those of O-WCO, and no thermal decomposition happened below 350 °C. This suggests that glycerol esterification can effectively improve WCO rejuvenators’ resistance to thermal–oxidative aging and improve the stability of their performance.
(2) The rheological performance test results indicate that D-RA is characterized by the lowest temperature sensitivity, a higher content of light components, superior high-temperature stability, and low-temperature crack resistance.
(3) Strong evidence for improving the durability of the recycled asphalt pavement is provided by the esterification treatment, which makes sure that the small molecular components added to the aged asphalt during the incorporation of WCO are more stable. This reduces the volatilization and oxidation during the thermal oxidation aging of the recycled asphalt.

## Figures and Tables

**Table 1:** Performance indexes of matrix asphalt.

Technical Indexes	Results	Test Methods
Penetration at 25 °C (0.1 mm)	65	ASTM D5 [31]
Softening point (°C)	50.0	ASTM D36 [32]
Ductility at 15 °C (cm)	>100	ASTM D113 [33]
Viscosity at 135 °C (Pa·s)	611.9	ASTM D4402 [34]
Flash point (°C)	290	ASTM D92 [35]

**Table 2:** Conventional physical attributes of WCO.

Technical Indexes	Results	Test Methods
Density at 15 °C (g/cm^3^)	0.91	ASTM D1298 [36]
Viscosity at 60 °C (mPa·s)	19	ASTM D445 [37]
Acid value (mgKOH/g)	65.28	ASTM D974 [38]
Iodine value (g/100 g)	131.13	ASTM D5558 [39]
Color	blackish brown	-

**Table 3:** Performance indicators of the virgin asphalt and the asphalt at different aging levels.

Type of Asphalt	Penetration (25 °C, 100 g, 5 s)/(0.1 mm)	Softening Point (Ring and Ball Method)/°C	Ductility (15 °C, 5 cm/min)/cm	Viscosity (mPa·s)
VA	57.5	50	>100	611.9
SA	49.4	55	7	826.4
LA	21.4	63	3.2	1130

**Table 4:** Names of rejuvenators and recycled asphalt with different acid value levels.

Name of Rejuvenator	Acid Value of WCO (mg KOH/g)	Name of Recycled Asphalt
O-WCO	60 ± 2	O-RA
M-WCO	30 ± 2	M-RA
D-WCO	4 ± 2	D-RA

**Table 5:** Reaction mechanism model fitting and kinetic parameterization of regenerant’s thermal decomposition process under nitrogen atmosphere.

Sample Name	Reaction Model	Simultaneous Equations	Correlation Coefficient	*E*	*A*	Δ*H*	Δ*G*	Δ*S*
D-WCO	D1	y = −3455.9x – 4.9965	0.9611	28,732	234	25,432	108,906	−210
O-WCO	F1	y = −601.9x – 12.071	0.9907	5004	0.0344	1577	118,599	−283

**Table 6:** Effect of aging on molecular weight distribution parameters of base and recycled asphalt.

Molecular Mass	Mn (g/mol)			Mw (g/mol)			PDI (Mw/Mn)		
Aging degree	Unaged	RTFO	PAV	Unaged	RTFO	PAV	Unaged	RTFO	PAV
Base asphalt	822	880	894	1704	2230	2570	2.07	2.48	2.59
Recycled asphalt	864	980	991	2111	2463	2647	2.44	2.51	2.67

## Data Availability

The data presented in this study are available on request from the corresponding author.
